# Rarity and nutrient acquisition relationships before and after prescribed burning in an Australian box-ironbark forest

**DOI:** 10.1093/aobpla/ply032

**Published:** 2018-05-16

**Authors:** John Patykowski, Matt Dell, Tricia Wevill, Maria Gibson

**Affiliations:** 1 Deakin University, Geelong, Australia; School of Life and Environmental Sciences, Centre for Integrative Ecology (Burwood Campus), Burwood, Victoria, Australia; 2 Ecology Australia Pty Ltd, Fairfield, Victoria, Australia

**Keywords:** Disturbance, functional rarity and uniqueness, hemiparasite, hyper-accumulator, mistletoe, nutrient cycling and resorption

## Abstract

Nutrient cycling is greatly influenced by dominant plants that contribute high amounts of leaf litter to soils; however, less-dominant and rare species can play keystone roles in nutrient cycling if they have unique nutrient acquisition traits and provide high-quality litter. In many parts of the world, wildfire is likely to become more frequent and intense under a changing climate. The effect this will have on plant rarity and on species with unique nutrient acquisition traits, and thus nutrient cycling, remains poorly understood. Working within an Australian box-ironbark forest, we determined if a relationship existed between species rarity and the uniqueness of their leaf nutrient profiles, and if this relationship changed after prescribed burning. We created an index of species rarity from a data set of woody perennial species abundance in areas before and after autumn or spring burns, or left unburnt. We created indices of uniqueness for the leaf nutrient profiles of 42 woody perennial species occurring in the ecosystem, based on amounts of six macronutrients and four micronutrients found in fresh and senesced leaves of each species. Five nutrient acquisition strategies (mycorrhizal, N-fixing, carnivorous, hemiparasitic and proteoid roots) were represented in the data set. There was no community-wide relationship between rarity and uniqueness of leaf nutrient profiles, and this did not change as a result of fire. However, two hemiparasitic species were relatively rare in the ecosystem studied, and differed greatly from other species due to high K and P in senesced leaves. Thus, some of the rarest species, such as hemiparasites, can be functionally unique. Understanding the functional characteristics of rare species is important so that unique functional contributors can be identified and conserved to prevent local extinction.

## Teaser

We examined the relationship between nutrient acquisition strategies, senesced leaf nutrient profiles and species rarity before and after prescribed burning in a nutrient-poor box-ironbark forest in southeastern Australia. While no community-wide relationship between rarity and uniqueness of leaf nutrient profiles was found, some of the rarest species were functionally unique. Two hemiparasitic species were relatively rare in the ecosystem studied, and differed greatly from other species due to high concentrations of phosphorous and potassium in senesced leaves. Our study highlights the importance of identifying and ultimately conserving species with unique traits to prevent loss of functional contributions.

## Introduction

The internal resorption and redeployment of nutrients from senescing leaves into newly developing organs are an important adaptation of plants to conserve nutrients (Aerts 1996; Wright *et al.* 2004), particularly for species in ecosystems with depauperate soils (Wright and Westoby 2003; Hayes *et al.* 2013). The movement of nutrients from deeper soil layers through plant roots, to above-ground plant parts and, ultimately, to surface soils through the decomposition of senesced leaves, influences the availability of nutrients along soil horizons (Jobbágy and Jackson 2001; Jobbágy and Jackson 2004), affecting community species richness (Shirima *et al.* 2016) and productivity (Aerts and Chapin 1999). Nutrient profiles of senesced leaves can be species-specific (Hättenschwiler *et al.* 2008); thus, the rate and volume that nutrients are returned to the soil in a community through litterfall are dependent on its constituent species (Cornwell *et al.* 2008; Hobbie 2015).

Generally, the most dominant species, in terms of biomass, have the greatest influence on ecosystem processes (Gaston 2011), and contribute most to soil nutrient returns through sheer volume of litterfall; however, less-dominant and even rare species have been found to play keystone roles in nutrient cycling in some nutrient-poor environments (Marsh *et al.* 2000; March and Watson 2010). In such cases, the less-dominant species have relatively unique nutrient acquisition strategies compared to other members of their community, enabling better access to nutrients, and a lesser requirement for resorption (Marsh *et al.* 2000; March and Watson 2010). The nutrient-rich litter they provide to otherwise nutrient-poor soils has a bottom-up effect on other ecosystem properties (Quested *et al.* 2005; Watson and Herring 2012; Fisher *et al.* 2013). Disturbance leading to the loss of such species can have profound ecological consequences.

Fire directly influences nutrient cycling in ecosystems by changing the availability and distribution of nutrients (Certini 2005), and indirectly influences nutrient cycling by shaping plant community composition (Bond and Keeley 2005). Fire is a disturbance process affecting many parts of the world (Krawchuk *et al.* 2009) but, as far as we know, the effect of fire on the presence of less-common species and their post-fire role in nutrient cycling has not been explored. The initial effect of fire on species abundance can result in four extreme outcomes along a continuum: (i) less-common species are promoted and dominant species are reduced; (ii) dominant species are promoted and less-common species are reduced; (iii) both dominant and less-common species are promoted; or (iv) both dominant and less-common species are reduced. Within these outcomes, less-common species could play similar functional roles to the dominant species and facilitate ecosystem recovery through provision of functional insurance (Yachi and Loreau 1999; Jain *et al.* 2014), or be functionally unique and play novel functional roles. Important functions could be lost should disturbance negatively impact the presence of rare or less-common species with unique traits (Violle *et al.* 2017). Of course, a likely impact of any disturbance is a combination of these outcomes. Understanding contributions to nutrient cycling at both the species and community level, and how these contributions change following disturbance, is important for understanding ecosystem dynamics, especially given recent work highlighting the disproportionately large contributions made by rare species towards functional richness in ecosystems around the world (Mouillot *et al.* 2013; Leitão *et al.* 2016; Umaña *et al.* 2017).

Patterns of nutrient content, nutrient acquisition strategy and changes in species frequency of occurrence following disturbance were explored using a temperate, evergreen, box-ironbark forest in southeastern Australia as a case study. Concentrations of six macro- and four micronutrients in fresh and senesced leaves (and thus proportional resorption) were measured for each of 42 box-ironbark species representing five nutrient acquisition strategy groups (one carnivorous, two hemiparasitic, two proteaceous, 12 N-fixing and 25 mycorrhizal species). Plant frequency of occurrence was quantified before and 3 years after experimental landscape-scale prescribed burning in autumn and spring. Based on recent evidence (Mouillot *et al.* 2013; Leitão *et al.* 2016), it was expected that a positive relationship would exist between species rarity and uniqueness of leaf nutrient profiles. We also expected that this relationship would change if fire promoted the abundance of rarer species, and reduced the abundance of common species, indicating novel post-fire roles for some species that were rare and functionally unique, and functional insurance roles for species that were rare and functionally redundant. Finally, we asked if species with similar nutrient acquisition strategies would be similar in their senesced leaf nutrient profiles, and in their proportional withdrawal of different nutrients from fresh leaves, compared with species employing different nutrient acquisition strategies. We compared nutrient concentrations in the upper and lower soil horizons of the study area to indicate which nutrients were most limiting in the system (Jobbágy and Jackson 2004), and thus which species are capable of making important contributions to soil nutrient pools through the quality of their litter.

## Methods

### Study area

Data were collected from within the Heathcote-Graytown-Rushworth forest, the largest patch of contiguous box-ironbark forest in Victoria, southeastern Australia (ECC 2001). Soils are nutrient-poor and typically shallow, stony and skeletal clay loams forming undulating hills and peneplains, 150–300 m in elevation (Douglas and Ferguson 1988). The climate of the region is temperate; mean daily maximum temperatures are warmest in January (29 °C) and coolest in July (12 °C) (Redesdale weather station ID # 088051; Bureau of Meteorology 2016). The wettest month is August (69 mm) and the driest month is February (31 mm); mean annual rainfall is 594 mm (Heathcote weather station ID # 088029; Bureau of Meteorology 2016).

The vegetation is sclerophyllous and characterized by an open canopy of *Eucalyptus* species to 20 m tall, and a sparse to well-developed understorey of small trees and shrubs. Most species in this forest exhibit adaptation to fire including strong resprouting ability and fire-cued seed germination (Tolsma *et al.* 2007). A variety of nutrient acquisition strategies exist, including symbiotic associations with mycorrhizal fungi and N-fixing bacteria, hemiparasitism, carnivory and proteaceous rooting; a typical phenomenon in low-nutrient areas (Lambers *et al.* 2010). The forests themselves are increasingly subject to small-scale patchy fire, historically from lightning strike and, more recently, from fuel reduction burning and deliberately lit fires (DSE 2003).

### Species frequency data

**Figure 1.**
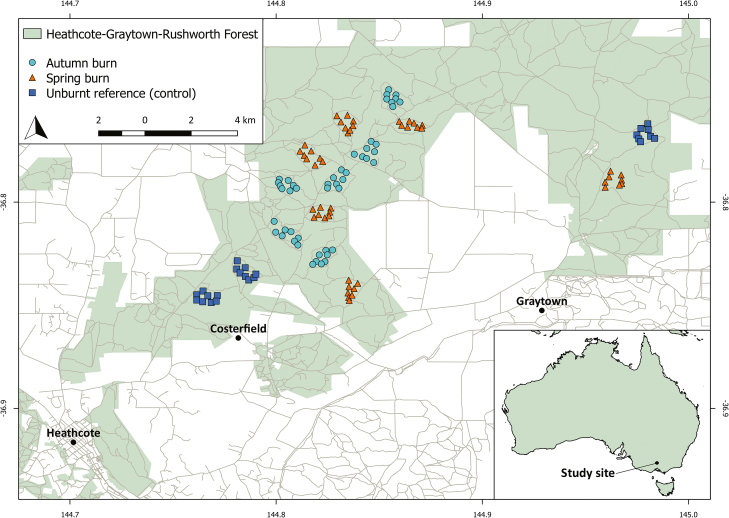
Location of study plots within the Heathcote-Graytown-Rushworth forest, southeastern Australia.

Pre- and post-fire species frequency data were collected as part of a broader study examining the effect of prescribed burns on ecological attributes within this forest (see Bennett *et al.* (2012) and Holland *et al.* (2017) for rationale and detailed methodology). Eight permanent 20 × 20 m plots were surveyed during spring (September–October) of 2010 for frequency of plant species, within each of 15 study areas within the Heathcote-Graytown-Rushworth forest (Fig. 1). All study areas were dominated by box-ironbark vegetation, and had similar underlying soils and geology (Douglas and Ferguson 1988). Each study area was a landscape ~70–120 ha in size and separated by a distance >500 m. Within each plot, five permanent 1 × 1 m quadrats were established at fixed locations. Species received a frequency score for each plot based on the number of 1 × 1 m quadrats that they occurred within, and were considered present if they were either growing within, or had foliage overhanging the boundary of the quadrat. Species received a score of 0.2 for each quadrat they occupied, to a maximum frequency score of 1. Species present in the 20 × 20 m plot but not in any of the five smaller quadrats received a frequency score of 0.2. Prescribed burns were conducted in the following autumn and spring of 2011, within six study areas per season; three study areas were left as unburnt, control areas. Burns in autumn were patchier than those in spring, when a greater extent of each survey plot was burnt (Holland *et al.* 2017), and it was expected that this would affect floristic composition differently. Each plot in each study area was then resurveyed in spring of 2013, 2 years after applying burn treatments, to understand how the vegetation responded to prescribed burns in autumn and prescribed burns in spring. Using the vegetation survey data, each species received a pre- and post-fire rarity score by multiplying the number of plots they occurred in by their mean abundance within the plots they occupied, divided by the maximum possible frequency score to place species on a rarity scale of 0–1, with zero representing absence.

### Leaf collections

Forty-two species were selected for leaf nutrient analysis, chosen to represent a range of rarity and nutrient acquisition strategies present in the community. We used published data and literature to assign species to their broad nutrient acquisition strategy group: carnivorous, hemiparasitic, mycorrhizal, N-fixing and proteaceous-root species **[see****Supporting Information—Table S1****]**. A minimum of 10 fresh leaves were collected from each of 20 plants per species. For widely distributed, common species we collected leaves from five plants within each of two unburnt study areas, and one autumn-burnt and one spring-burnt study area. For sparsely distributed and rarer species, leaf collection was opportunistic and occurred throughout the study areas where they were known to be present. Aerial hemiparasites were collected from the same species of host, and where more than one individual was parasitizing a host, samples from only one plant were collected. As nutrient concentrations are highly dependent on leaf age (Wright and Westoby 2003), we collected fully mature and fully expanded sun leaves from the most recent year’s growth. Collections of fresh leaves were made between August and September (late winter to early spring) of 2014. Senesced leaves were collected in early summer of 2014 and were considered those with a fully formed abscission layer preventing further nutrient withdrawal. These were readily identifiable as the leaves were often yellow-brown in colour and easily detached from the plant. Leaves already in the litter layer were not collected. Senesced leaves were often scarce, so a minimum of 50 senesced leaves were collected in total, from a minimum of 20 plants, per species. This ensured a representative sample of the population could be obtained and sufficient material was available for nutrient analysis. All leaves were collected in the field wearing nitrile gloves, and were gently wiped with damp paper towel to remove potential surface contaminants such as dust. Following collection, leaves were dried at 60 °C in a fan-forced oven for 72 h, until dry. An equal amount of leaf material was weighed from each plant collected for a species. This was finely ground into a homogenized powder using a coffee grinder, to produce a representative sample of fresh and senesced material per species.

### Soil sampling

Soil samples were collected from three plots within each of 15 study areas to assess soil nutrient concentrations and determine limiting nutrients. Collections were undertaken in November of 2014, ~3 years after the application of burn treatments. Samples of the topsoil (0–20 cm depth) and of deeper soil (50–60 cm depth) were collected to capture potential change in nutrient status between soil horizons (Wigley *et al.* 2013). Top- and subsoil samples were collected at each of three random locations per plot. Each replicate was dried in a fan-forced oven at 60 °C for 72 h, until dry. Each sample was sieved to remove debris and rock, and ground to a fine powder using a coffee grinder. An equal amount of soil was then weighed from each sample and bulked to give a representative sample of the top- and subsoil within each plot.

### Nutrient concentration

Soil and leaf samples were analysed for six macronutrients (nitrogen [N], potassium [K], calcium [Ca], magnesium [Mg], phosphorus [P] and sulphur [S]) and four micronutrients (copper [Cu], manganese [Mn], boron [B] and zinc [Zn]) by Environmental and Analytical Laboratories (EAL; Charles Sturt University, Wagga Wagga, NSW, Australia, eal@cus.edu.au). Inductively coupled plasma mass spectrometry was used to measure the concentration of all nutrients except total N, using standard American Public Health Association methods (APHA 3030 E and 3120 B; http://standardmethods.org/). For total N, the Dumas method of high temperature combustion (Method 7A5) was used (Rayment and Lyons 2011). Proportional resorption of each nutrient was calculated on a mass basis for each species by dividing the nutrient content of senesced leaves by the nutrient content of fresh leaves.

### Data analysis

To determine the uniqueness of species nutrient profiles, we created a similarity matrix by scaling nutrient variables (subtracting the mean and dividing by the standard deviation) and using PRIMER version 7 (PRIMER-E Ltd, Plymouth, UK) to calculate Euclidean distances between species pairs. We then ranked species from most to least similar, such that each species pair received a rank. The mean rank of each species was then calculated and divided by the maximum possible mean rank (the maximum mean rank representing a species least similar to the rest of the community) to place species on a scale from 0 to 1 (most to least similar to the rest of the community).

The relationship between the uniqueness of species’ senesced leaf nutrient profiles and species’ rarity scores was analysed using linear models in R version 3.3.1 (R Core Team 2016). Models were produced for senesced leaf nutrient profiles, and for proportional resorption, as functions of species pre- and post-fire rarity throughout all study areas, as well as separately within the unburnt, autumn, and spring-burn study areas.

Principal component analysis (PCA) was conducted in PRIMER on standardized senesced leaf nutrient values, and also proportional resorption values, to determine which nutrients were important in separating and clustering species based on their similarity, and to identify emerging patterns based on species nutrient acquisition strategies.

Differences in nutrient concentrations between soil depths, among burn treatments, and interactive effects of soil depth and burn treatment on soil nutrient concentrations, were tested using analysis of variance in R.

## Results

### Uniqueness of leaf nutrient profiles and rarity

**Figure 2.**
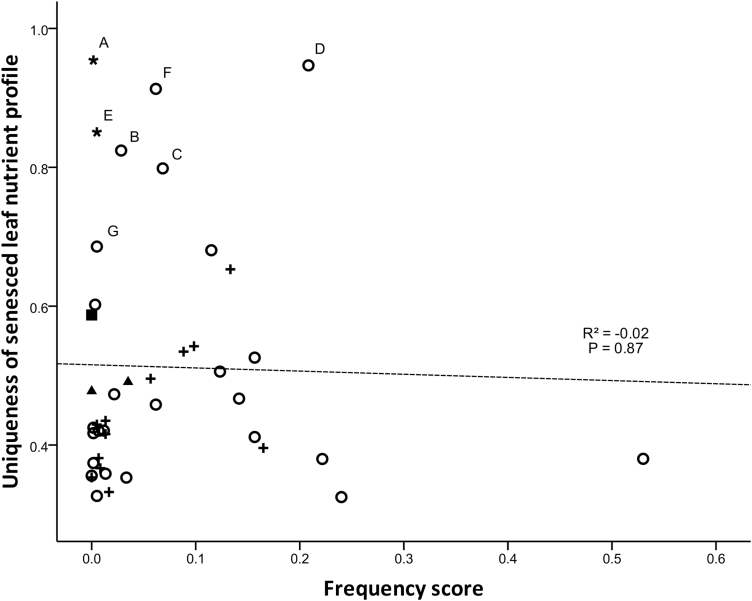
Relationship between species pre-fire frequency and uniqueness of their senesced leaf nutrient profile. Letters represent species identified as important contributors of sampled nutrients (outlier species) in PCA. A = *Amyema miquelii*; B = *Brunonia australis*; C = *Bursaria spinosa*; D = *Cassinia arcuata*; E = *Exocarpos cupressiformis*; F = *Ozothamnus obcordatus*; G = *Prostanthera denticulata*. Symbols represent species’ nutrient acquisition strategy (filled square = carnivorous; filled triangle = proteaceous roots; open circle = mycorrhizal; + = N-fixing rhizobium bacteria; black asterisk = hemiparasitic).

**Figure 3.**
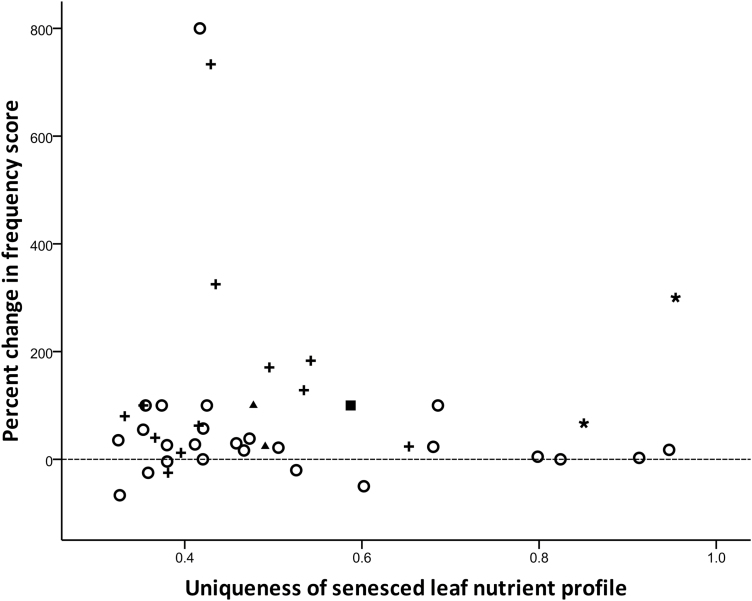
Uniqueness of leaf nutrient profile and percent change in species frequency score in the landscape following disturbance. Dashed line represents zero change in frequency score before and after prescribed burning as a disturbance. Symbols represent species’ nutrient acquisition strategy (filled square = carnivorous; filled triangle = proteaceous roots; open circle = mycorrhizal; + = N-fixing rhizobium bacteria; black asterisk = hemiparasitic).

There was no relationship between species frequency score (either before or after fire) and the overall nutrient profile in senesced leaves, or in the proportional resorption of nutrients (*P* > 0.05; see **Supporting Information—Table S2**). A number of rarer species were functionally similar to dominant members of the community; however, some less-common species possessed leaf nutrient profiles with a high level of uniqueness, and these species may individually or collectively be important for their nutrient contributions to soils (Fig. 2). Most species became more frequent throughout the study areas following prescribed burn treatments; those that decreased in frequency were functionally similar in leaf nutrient characteristics to species that increased in frequency (Fig. 3).

### Nutrient acquisition strategies and leaf nutrient profiles

**Figure 4.**
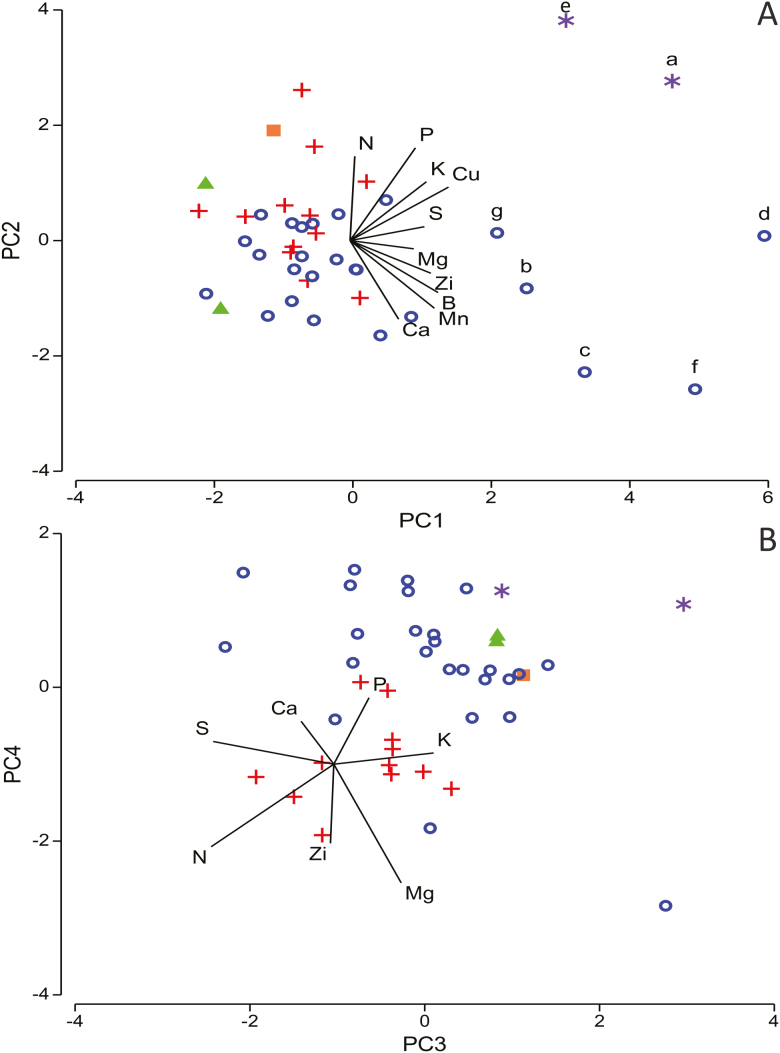
PCA ordination of 42 box-ironbark species by similarity of senesced leaf nutrient content for six macro- and four micronutrients. PC1 and PC2 (panel A) and PC3 and PC4 (panel B) are displayed (explaining 53.1 and 23 % of variation in the data, respectively), showing important contributors of sampled nutrients (outlier species). Length of line indicates relative strength of the influence of the nutrient. Symbols represent species’ nutrient acquisition strategy (filled square = carnivorous; filled triangle = proteaceous roots; open circle = mycorrhizal; + = N-fixing rhizobium bacteria; black asterisk = hemiparasitic). Important contributors of sampled nutrients (outlier species): a = *Amyema miquelii*; b = *Brunonia australis*; c = *Bursaria spinosa*; d = *Cassinia arcuata*; e = *Exocarpos cupressiformis*; f = *Ozothamnus obcordatus*; g = *Prostanthera denticulata*.

Two hemiparasitic species*—Amyema miquelii* and *Exocarpos cupressiformis*—separated from all other species in the PCA plot comparing senesced leaf nutrient content (Fig. 4), with the first two principal components (PCs) explaining 53.1 % of variation among species **[see****Supporting Information—Table S3****]**. Concentrations of P, K and Cu were substantially higher in the senesced leaves of these hemiparasites than the community average for these nutrient concentrations (Table 1), and were the most influential nutrients in causing separation of these species in the PCA plot (Fig. 4). Five mycorrhizal species also separated out from the main group, predominantly due to their high concentrations of Mn, Zn and Cu (Table 1). Clustering of N-fixing species occurred when plotting PC3 and PC4 (explaining 23 % of the variation among species; Fig. 4). Concentrations of N in the senesced leaves of N-fixing species were generally higher than concentrations found for species with other nutrient acquisition strategies **[see****Supporting Information—Table S1****]**, and this nutrient, along with Mg, was greatly responsible for the clustering observed in Fig. 4.

**Table 1.** Leaf nutrient concentrations (mg kg^−1^) for box-ironbark species identified as important contributors of sampled nutrients through PCA (Fig. 3) for senesced leaf nutrient concentration. Nutrient concentration and proportional resorption of nutrients for all 42 species sampled are provided in **Supporting Information—Tables S1 and S2**. Figures in bold indicate values that are greater than double the community mean, which is derived from all 42 species sampled in this study. Nutrient acquisition strategy (NAS): H = hemiparasitic; M = mycorrhizal.

Species	NAS	N	K	Ca	Mg	P	S	B	Mn	Zn	Cu
*Amyema miquelii*	H	5940	**28900**	6940	3250	**553**	1510	94	380	24.6	**14.4**
*Brunonia australis*	M	3620	**10300**	6860	**5440**	210	606	56	408	**130**	7.4
*Bursaria spinosa*	M	7420	4800	9360	3860	226	1260	91	**1040**	**140**	4.3
*Cassinia arcuata*	M	7500	6030	5880	2690	406	**2070**	**150**	**1040**	**99.6**	**22.1**
*Exocarpos cupressiformis*	H	10200	**12500**	6020	3140	**841**	1500	36	429	19.2	**14.3**
*Ozothamnus obcordatus*	M	7060	5340	11800	2100	271	**2410**	**118**	**1702**	**68.5**	**10**
*Prostanthera denticulata*	M	10700	2840	4820	1500	342	1370	87	**751**	**103**	8.4
**Community mean**		**7513**	**3726**	**6424**	**2088**	**224**	**1104**	**55**	**365**	**27**	**5**

**Figure 5.**
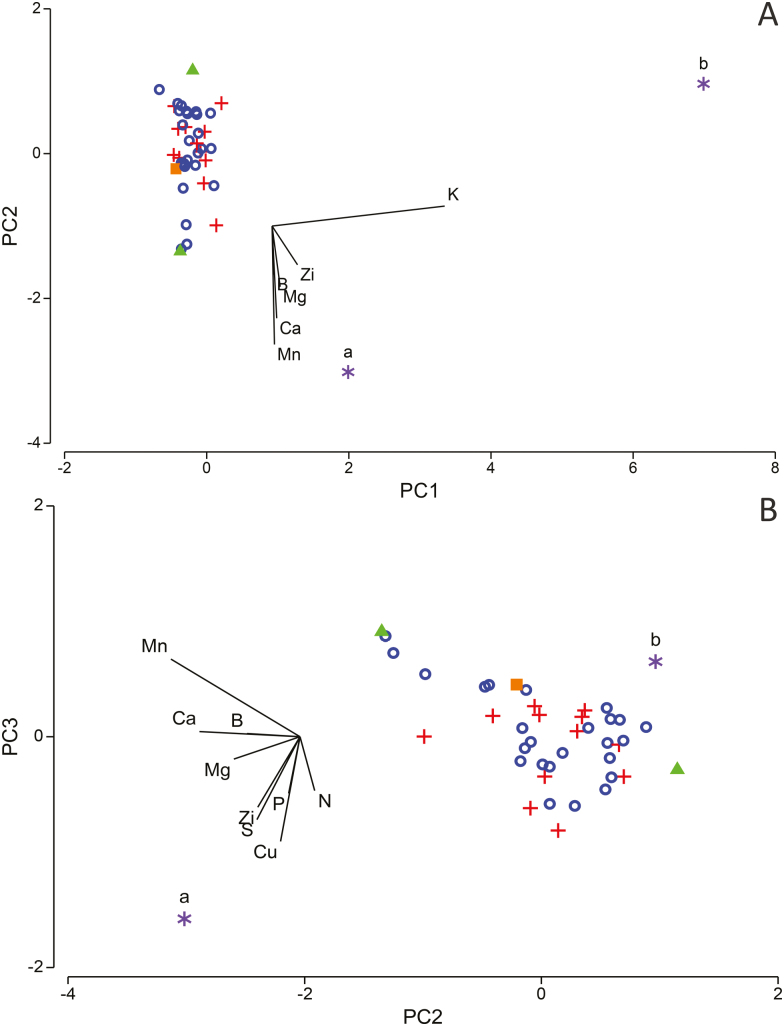
PCA ordination of 42 box-ironbark species by similarity of resorption of six macro- and four micronutrients prior to leaf drop. PC1 and PC2 (panel A) and PC2 and PC3 (panel B) are displayed (explaining 78.3 and 32.5 % of variation in the data, respectively). Length of line indicates relative strength of the influence of the nutrient. Symbols represent species’ nutrient acquisition strategy (filled square = carnivorous; filled triangle = proteaceous roots; ○open circle = mycorrhizal; + = N-fixing rhizobium bacteria; black asterisk = hemiparasitic). Important contributors of sampled nutrients (outlier species): a = *Amyema miquelii*; b = *Exocarpos cupressiformis*.

When comparing proportional withdrawal of nutrients from senesced leaves, both hemiparasitic species separated from the main cluster of species. No clear separation occurred for any other nutrient acquisition strategy (Fig. 5). The concentration of K in the senesced leaves of *E. cupressiformis* was 671 % higher than in fresh leaves. This influenced its position on the PCA plot relative to other species (Fig. 5) when considering the first two PCs (explaining 78.3 % of variation among species; **see****Supporting Information—Table S3**). When considering PC2 and PC3, which explained 32.5 % of the variation in the data and where K was less influential, *A. miquelii* separated from all other species (Fig. 5). The concentration of all nutrients was higher in the senesced leaves of *A. miquelii* than for any other species (except for N, which it withdrew similarly as all other species in the community), which influenced its position in the plot. Notably, all species except *A. miquelii* withdrew P. Values of macro- and micronutrient concentration in fresh and senesced leaves for each species are provided in **Supporting Information—Table S1**.

### Soil nutrients and fire

**Figure 6.**
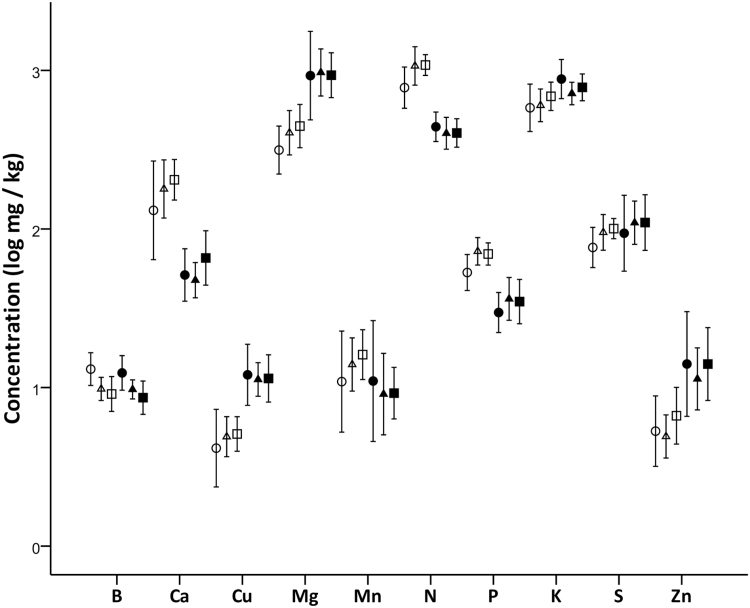
Concentration (log mg kg^−1^ ± SE) of key macro- and micronutrients in upper (0–20 cm; open symbols) and lower (50–70 cm; closed symbols) soil horizons in a box-ironbark forest in southeastern Australia. Samples were collected 3 years after landscape-scale prescribed burn treatments. Circles = unburnt reference landscapes; triangles = autumn burn landscapes; squares = spring burn landscapes.

Soil nutrient profiles generally differed between the upper (0–20 cm) and lower (50–70 cm) horizons (Table 2). Soil nutrient profiles did not differ between burn treatments 3 years post-fire, except for boron, which was higher in unburnt study areas (Fig. 6); there was no interacting effect of season of burn and sample depth on nutrient concentrations. Three nutrients were found in higher concentrations in the upper soil horizon (Ca, N and P), and four were more concentrated in the lower soil horizon (Cu, Mg, K and Zn; Table 2 and Fig. 6). Mean nutrient concentrations for each nutrient, by depth and burn treatment, are provided in **Supporting Information—Table S4**.

**Table 2.** Difference in concentration of nutrients between soil depths (0–20 cm, 50–70 cm), among burn treatments (unburnt control, autumn burn, spring burn) and their interaction, in a box-ironbark forest. Soil samples were collected 3 years after landscape-scale prescribed burn treatments. Significant results from ANOVA are highlighted in bold.

Nutrient	*F*	df	*P*	Nutrient	*F*	df	*P*
Boron				Nitrogen (total)			
Depth	0.19	1,1	0.66	Depth	**78.89**	**1,1**	**<0.001**
Season	**4.97**	**1,2**	**0.009**	Season	0.52	1,2	0.59
Depth * season	0.04	1,2	0.96	Depth * season	1.68	1,2	0.19
Calcium				Phosphorus			
Depth	**4.90**	**1,1**	**<0.001**	Depth	**35.79**	**1,1**	**<0.001**
Season	0.16	1,2	0.19	Season	1.67	1,2	0.19
Depth * season	0.04	1,2	0.63	Depth * season	0.09	1,2	0.91
Copper				Potassium			
Depth	**47.11**	**1,1**	**<0.001**	Depth	**7.03**	**1,1**	**0.01**
Season	0.10	1,2	0.90	Season	0.70	1,2	0.50
Depth * season	0.32	1,2	0.73	Depth * season	0.793	1,2	0.46
Magnesium				Sulphur			
Depth	**37.65**	**1,1**	**<0.001**	Depth	1.22	1,1	0.27
Season	0.47	1,2	0.64	Season	0.85	1,2	0.43
Depth * season	0.41	1,2	0.67	Depth * season	0.06	1,2	0.94
Manganese				Zinc			
Depth	2.57	1,1	0.11	Depth	**19.70**	**1,1**	**<0.001**
Season	0.11	1,2	0.90	Season	0.80	1,2	0.45
Depth * season	0.59	1,2	0.56	Depth * season	0.10	1,2	0.90

## Discussion

We did not observe a linear relationship between species rarity and the uniqueness of leaf nutrient profiles, and this relationship did not change as the result of a prescribed fire. However, we found that a number of rarer species possessed relatively unique leaf nutrient profiles, and that nutrient uptake strategy could indicate similarity among species nutrient profiles.

### Uniqueness of leaf nutrient profiles and rarity

Studies exploring relationships between rarity and functional uniqueness generally find weak to moderate, positive trends (e.g. Mouillot *et al.* 2013). This includes greater than expected functional uniqueness of rare species relative to common species (Leitão *et al.* 2016) and, thus, their roles in ecosystem functioning are of greater importance than would be expected based on abundance. These studies incorporated broader suites of functional traits in their analysis than our own. A positive relationship between rarity and functional uniqueness may exist within the ecosystem we studied, but it does not occur when looking at leaf nutrient content alone.

It is not surprising that the relationship between rarity and uniqueness of leaf nutrient profiles did not change as a result of fire in the forest we studied. In most fire-tolerant ecosystems around the world, a proportion of the species are adapted to resprout after burning, and indeed, no substantial change in abundance was observed among species in this study, many of which are able to resprout (Tolsma *et al.* 2007). The effect of a disturbance that is more intense than an autumn or spring burn (i.e. a summer burn that scorches or burns the canopy) may have wider implications for the persistence of some species. For example, *A. miquelii* (an aerial hemiparasite with a unique senesced leaf nutrient profile) does not have fire-stimulated germination and recovers weakly, if at all, after burning (Kelly *et al.* 1997), should a fire reach it in the canopy. Recolonization of burnt sites with *A. miquelii* would require seed dispersal from surrounding populations in the landscape. Consequently, the extent and severity of the disturbance becomes an important factor governing the rate that a unique functional role is restored.

### Nutrient acquisition strategies and leaf nutrient profiles

The two hemiparasites (*A. miquelii* and *E. cupressiformis*) present in the study areas had unique leaf nutrient profiles because of their concentrations of K and P (up to 7.75 times higher than the community mean for K, and 3.75 times for P). High concentrations of K have previously been noted in the leaves of the aerial hemiparasite *A. miquelii* (March and Watson 2010). We add to this by demonstrating the root hemiparasite *E. cupressiformis* also has high levels of K in senesced leaves, and had the highest levels of P in the community. Hemiparasitic plants are important contributors to soil nutrient cycles in ecosystems around the world, through litterfall rich in nutrients such as K and P (Quested *et al.* 2003; March and Watson 2010; Ndagurwa *et al.* 2014; Scalon *et al.* 2017). Litterfall from hemiparasitic species has positive effects on community productivity (March and Watson 2007; Spasojevic and Suding 2011; Fisher *et al.* 2013) and bottom-up effects on diversity (Watson 2002), leading some to describe them as a keystone resource (Watson and Herring 2012).

It is concerning that the two hemiparasitic species observed in our study were among the least frequently encountered species. Should a disturbance cause the loss of the hemiparasitic species or their hosts, their functional contribution will be lost as well. *Amyema miquelii* is fire-sensitive and does not resprout after being burnt (Kelly *et al.* 1997), whereas *E. cupressiformis* is known to resprout after fire (Kubiak 2009). Hemiparasites have a nearly cosmopolitan distribution and often very specific germination requirements (Watson 2001). Understanding hemiparasite and host tolerances to stress and responses to disturbance is necessary to highlight which ecosystems are vulnerable to loss of this functional group in the future, and what processes may cause this loss.

Nitrogen-fixing species contribute N to community nutrient pools through below-ground fixation of N (Adams and Attiwill 1984a), and leaf-fall. Resorption of N is generally lower in N-fixing species than those that do not fix nitrogen (Killingbeck 1996) and, indeed, we found that N-fixing species generally had higher concentrations of N in senesced leaves than other members of the community. Many nitrogen-fixing species (e.g. members of Fabaceae) from fire-prone environments use fire as a cue for mass germination (Adams and Attiwill 1984b). Such changes to the abundance of N-fixing species can confer an enhanced ecosystem role in the years following fire, particularly for providing N nutrition to non-N-fixing species (Pfautsch *et al.* 2009). Although we observed a small change in species abundance following disturbance, several N-fixing species were relatively common even before prescribed burning (Fig. 2). Further investigation of the ecophysiological tolerances to disturbances (e.g. fire, drought, flood, temperature, disease) possessed by the N-fixing species observed in this study is warranted to determine how stable the process of nitrogen fixation is in this ecosystem.

There was no clear segregation of mycorrhizal species in our analysis. We opted for a broad mycorrhizal category and did not split species into different associations (e.g. into ectomycorrhizas, ericoid mycorrhizas or vesicular-arbuscular mycorrhizas), as they are uncharacterized for many species in this community. However, mycorrhizal associations can offer plants enhanced access to different nutrients, depending on the type of association and species (Marschner and Dell 1994; Read and Perez-Moreno 2003), and affect the functional traits of plants. For example, ectomycorrhizal fungi are able to breakdown organic matter, whereas vesicular-arbuscular mycorrhiza predominantly scavenge inorganic nutrients that are released by microbes (Read and Perez-Moreno 2003; Richard *et al.* 2013). Thus, plants with vesicular-arbuscular mycorrhiza often have leaf litter that is more easily broken down by microbial action than plants with ectomycorrhizal associations. Studies investigating such differences between co-occurring species are few (Richard *et al.* 2013), but the implication of differences among mycorrhizal associations, and their functional roles in nutrient cycling, is worthy of further attention.

The functional significance of species with high concentrations of micronutrients in their leaves remains to be explored, as these nutrients, although essential for plant physiology, are usually less limiting to plants than macronutrients and are thus less studied (Marschner and Marschner 2011). Several species in our study stood out for the high concentrations of micronutrients (B, Cu, Mn and Zn) in their senesced leaves. Three of the more common species (*Cassinia arcuata*, *Ozothamnus obcordatus* and *Bursaria spinosa*) had high levels of Mn and Zn in their leaves; up to 4.7 and 5.2 times the community average, respectively. In plantations, species of *Eucalyptus* (which also forms the canopy of the ecosystem we studied) have been shown to translocate Mn from lower to upper soil layers and change soil chemistry (Jobbágy and Jackson 2004). The apparent hyper-accumulation of Mn in *B. spinosa*, *C. arcuata* and *O. obcordatus* (soil concentrations were on average 20 mg kg^−1^ and senesced leaves were up to 1700 mg kg^−1^) could be a physiologic adaptation to cope with Mn accumulation in soils through *Eucalyptus* leaf litter, and should be explored.

### Soil nutrients

Over 3 years had passed between burn treatments and soil sampling in this study, so overall similarity in soil nutrient levels between burnt and unburnt areas was unsurprising. An ephemeral increase in soil nutrients is a well-known consequence of fire, resulting from the combustion of plant material. Often, soil nutrients quickly return to pre-fire levels, within several months (Adams and Boyle 1980; Macadam 1987; Weston and Attiwill 1990; Tomkins *et al.* 1991) to several years (Simard *et al.* 2001), as they are quickly taken up by living cells or lost through wind and water erosion, or soil leaching (Thomas *et al.* 1999; Certini 2005).

Soil nutrients that are most strongly cycled through the community tend to aggregate in the upper layers of the soil. This is because they are either translocated and quickly absorbed by plant roots before they can leach into lower soil layers (Jobbágy and Jackson 2001), or because some dominant species in the community have a high requirement for a mineral and thus transform the deposition of nutrients in soil layers (Jobbágy and Jackson 2004). We found that Ca, N and P were concentrated in the upper layers. Calcium deficiency in soils is generally rare in natural systems (White and Broadley 2003), and although plants generally have a high requirement for Ca (Marschner and Marschner 2011), it is likely Ca is in ample supply in this system and is less limiting. Deficiency in soil N and P is common in nutrient-poor systems around the world; thus, species contributing litter rich in these nutrients likely play important roles in community productivity and diversity, particularly for species with shallow root systems. Indeed, all but three species withdrew N from senescing leaves, with a community mean of 41.7 % withdrawal. Three species did not withdraw N—these species were small to prostrate shrubs with leaves that, when fallen, appear to become trapped and accumulate underneath the plant. Further study is required to understand if there is adaptive significance to this trait.

## Conclusions

Community-wide relationships between rarity and the uniqueness of species’ leaf nutrient profiles do not occur in Australian box-ironbark forest. However, some of the rarest species (those with low abundance) with uncommon strategies for nutrient acquisition (such as hemiparasitism) can support some of the most unique leaf nutrient profiles. Species capable of making unique contributions to soil nutrient pools deserve greater attention, so that their functional roles can be better understood and conserved.

## Supporting Information

The following additional information is available in the online version of this article—

**Table S1.** Concentration of macronutrients (mg kg^−1^) in fresh (F) and senesced (S) leaves of species growing in a box-ironbark forest in southeastern Australia, as well as proportional resorption of nutrients (%).

**Table S2.** The relationship between species rarity and the uniqueness of leaf nutrient profile in senesced leaves and in proportional resorption of nutrients from leaves for 42 species from a box-ironbark forest in southeastern Australia. Linear models were produced with respect to species frequency within 15 study areas (all sites) in 2010 (pre-burn) and 2013 (post-burn), as well as separately for sites which experienced a burn treatment in 2010 spring autumn, spring, or left unburnt.

**Table S3.** Eigenvector scores and percent variation explained by five principal components (PCs) for the similarity of leaf nutrient profiles, and proportional resorption, among 42 evergreen species in a box-ironbark forest in southeast Australia. All 10 macro- and micronutrients sampled are included in the principal component analysis (PCA) as eigenvectors. Proportional resorption was calculated as the ratio of nutrients in fresh and senesced leaves.

**Table S4.** Mean (±SD) concentrations of nutrients (mg kg^−1^) in soil samples from a box-ironbark forest in southeastern Australia, collected 3 years after landscape-scale experimental prescribed burn treatments in autumn, spring or left as unburnt reference study areas. Samples were taken from upper (0–20 cm) and lower (50–70 cm) soil profiles.

Supplementary Information

## Contributions by the Authors

J.P., M.D., T.W. and M.G. conceived ideas and designed methodology; J.P. coordinated all field activities and collected the data; J.P. analysed the data; J.P., M.D., T.W. and M.G. wrote the manuscript. All authors contributed critically to the draft and gave final approval for publication.

## Conflict of Interest

None declared.
