# Supplementary material for: Rarity and nutrient acquisition relationships before and after prescribed burning in an Australian box-ironbark forest
Source: AoB Plants. 2018 May 16;10(3):ply032. doi: 10.1093/aobpla/ply032 (PMC6007787; doi:10.1093/aobpla/ply032)
Supplement: Supplementary Information [file ply032_suppl_supplementary_information.docx]

**Table S1.** Concentration of macronutrients (mg^-1^/kg^-1^) in fresh (F) and senesced (S) leaves of species growing in a box-ironbark forest in southeastern Australia, as well as proportional resorption of nutrients (%). NAS = Nutrient acquisition strategy assigned to each species (N = N-fixing, M = mycorrhizal, H = hemiparasite, C = carnivorous, Pt = proteaceous roots); references are provided as a footnote. Where no nutrient acquisition strategy could be cited, species were assumed to be mycorrhizal.

| **Species** | **NAS** | **Nitrogen (N)** | | | **Potassium (K)** | | | **Calcium (Ca)** | | | **Magnesium (Mg)** | | | **Phosphorus (P)** | | | **Sulphur (S)** | | |
| --- | --- | --- | --- | --- | --- | --- | --- | --- | --- | --- | --- | --- | --- | --- | --- | --- | --- | --- | --- |
|  |  | **F** | **S** | **%** | **F** | **S** | **%** | **F** | **S** | **%** | **F** | **S** | **%** | **F** | **S** | **%** | **F** | **S** | **%** |
| *Acacia acinacea* | N^1,2^ | 25000 | 18300 | -26.8 | 4630 | 4160 | -10.2 | 2520 | 2010 | -20.2 | 2650 | 2530 | -4.5 | 499 | 217 | -56.5 | 1450 | 1150 | -20.7 |
| *Acacia aspera* | N^1,2^ | 16900 | 10800 | -36.1 | 3620 | 2580 | -28.7 | 3920 | 4600 | 17.3 | 1990 | 2010 | 1.0 | 486 | 170 | -65.0 | 1420 | 998 | -29.7 |
| *Acacia genistifolia* | N^1,2^ | 17800 | 7290 | -59.0 | 8250 | 2840 | -65.6 | 7460 | 7810 | 4.7 | 3160 | 3550 | 12.3 | 511 | 50 | -90.2 | 2480 | 1650 | -33.5 |
| *Acacia gunnii* | N^1,2^ | 14900 | 9430 | -36.7 | 4670 | 2360 | -49.5 | 4210 | 5480 | 30.2 | 2280 | 2860 | 25.4 | 446 | 118 | -73.5 | 1030 | 704 | -31.7 |
| *Acacia montana* | N^1,2^ | 19400 | 8970 | -53.8 | 4320 | 2740 | -36.6 | 4640 | 9500 | 104.7 | 1540 | 1700 | 10.4 | 614 | 134 | -78.2 | 1500 | 1180 | -21.3 |
| *Acacia paradoxa* | N^1,2^ | 20400 | 13500 | -33.8 | 4990 | 2370 | -52.5 | 7340 | 6790 | -7.5 | 2700 | 2490 | -7.8 | 519 | 136 | -73.8 | 1840 | 1250 | -32.1 |
| *Acacia pycnantha* | N^1,2^ | 21200 | 10000 | -52.8 | 7270 | 1990 | -72.6 | 4640 | 7990 | 72.2 | 2890 | 2660 | -8.0 | 740 | 72 | -90.3 | 1790 | 929 | -48.1 |
| *Acrotriche serrulata* | M^1^ | 8400 | 8560 | 1.9 | 4220 | 2210 | -47.6 | 11400 | 9130 | -19.9 | 1240 | 1100 | -11.3 | 494 | 307 | -37.9 | 1420 | 1190 | -16.2 |
| *Amyema miquelii* | H^1^ | 8360 | 5940 | -28.9 | 14500 | 28900 | 99.3 | 2470 | 6940 | 181.0 | 1340 | 3250 | 142.5 | 451 | 553 | 22.6 | 603 | 1510 | 150.4 |
| *Astroloma humifusum* | M^1^ | 8380 | 8450 | 0.8 | 4260 | 1840 | -56.8 | 11400 | 10400 | -8.8 | 985 | 887 | -9.9 | 460 | 317 | -31.1 | 1390 | 1030 | -25.9 |
| *Boronia anemonifolia* | M^3^ | 11000 | 5540 | -49.6 | 7100 | 3590 | -49.4 | 7130 | 8630 | 21.0 | 3030 | 3180 | 5.0 | 739 | 230 | -68.9 | 1400 | 1190 | -15.0 |
| *Brachyloma daphnoides* | M^1^ | 10500 | 4220 | -59.8 | 3460 | 2720 | -21.4 | 4200 | 4030 | -4.0 | 1920 | 1700 | -11.5 | 547 | 124 | -77.3 | 1130 | 929 | -17.8 |
| *Brunonia australis* | M^4^ | 24800 | 3620 | -85.4 | 28600 | 10300 | -64.0 | 6970 | 6860 | -1.6 | 5030 | 5440 | 8.2 | 1070 | 210 | -80.4 | 1630 | 606 | -62.8 |
| *Bursaria spinosa* | M | 13200 | 7420 | -43.8 | 8750 | 4800 | -45.1 | 8090 | 9360 | 15.7 | 3970 | 3860 | -2.8 | 734 | 226 | -69.2 | 1360 | 1260 | -7.4 |
| *Cassinia arcuata* | M^5,6^ | 17300 | 7500 | -56.6 | 17300 | 6030 | -65.1 | 4130 | 5880 | 42.4 | 2840 | 2690 | -5.3 | 1110 | 406 | -63.4 | 2170 | 2070 | -4.6 |
| *Daviesia leptophylla* | N^1,2,4^ | 18100 | 10200 | -43.6 | 6840 | 2680 | -60.8 | 1690 | 2770 | 63.9 | 2110 | 1830 | -13.3 | 431 | 67 | -84.5 | 1250 | 755 | -39.6 |
| *Daviesia ulicifolia* | N^1,2,4^ | 17300 | 15000 | -13.3 | 4810 | 1960 | -59.3 | 2790 | 3630 | 30.1 | 1970 | 2270 | 15.2 | 375 | 134 | -64.3 | 1430 | 1380 | -3.5 |
| *Dianella admixta* | M^7^ | 10800 | 5520 | -48.9 | 9580 | 1580 | -83.5 | 5640 | 5920 | 5.0 | 1190 | 825 | -30.7 | 688 | 207 | -69.9 | 1580 | 823 | -47.9 |
| *Dillwynia sericea* | N^1,4^ | 13800 | 9460 | -31.4 | 4450 | 1290 | -71.0 | 4860 | 4270 | -12.1 | 1330 | 876 | -34.1 | 370 | 108 | -70.8 | 1090 | 731 | -32.9 |
| *Drosera peltata* | C^1,3^ | 15400 | 7960 | -48.3 | 7980 | 2670 | -66.5 | 2290 | 3080 | 34.5 | 2020 | 2150 | 6.4 | 1930 | 525 | -72.8 | 1090 | 554 | -49.2 |
| *Eucalyptus goniocalyx* | M^3,4^ | 11000 | 4130 | -62.5 | 4860 | 2580 | -46.9 | 8350 | 7310 | -12.5 | 2180 | 2390 | 9.6 | 538 | 150 | -72.1 | 881 | 522 | -40.7 |
| *Eucalyptus macrorhyncha* | M^3,4^ | 9650 | 4140 | -57.1 | 4390 | 1540 | -64.9 | 3860 | 7400 | 91.7 | 2180 | 2430 | 11.5 | 488 | 118 | -75.8 | 763 | 450 | -41.0 |
| *Eucalyptus melliodora* | M^3,4^ | 13600 | 7220 | -46.9 | 8850 | 3710 | -58.1 | 6880 | 8000 | 16.3 | 2320 | 1960 | -15.5 | 834 | 234 | -71.9 | 1440 | 1040 | -27.8 |
| *Eucalyptus microcarpa* | M^3,4^ | 11200 | 7230 | -35.4 | 6620 | 2630 | -60.3 | 4610 | 7480 | 62.3 | 3010 | 2400 | -20.3 | 567 | 246 | -56.6 | 1050 | 804 | -23.4 |
| *Eucalyptus polyanthemos* | M^3,4^ | 13500 | 6210 | -54.0 | 9970 | 4360 | -56.3 | 5340 | 11000 | 106.0 | 2180 | 2420 | 11.0 | 661 | 160 | -75.8 | 1180 | 957 | -18.9 |
| *Eucalyptus tricarpa* | M^3,4^ | 11900 | 6620 | -44.4 | 5330 | 2460 | -53.8 | 4560 | 3460 | -24.1 | 1820 | 1500 | -17.6 | 565 | 238 | -57.9 | 1120 | 888 | -20.7 |

**Table S1 (contd.).** Concentration of macronutrients (mg^-1^/kg^-1^) in fresh (F) and senesced (S) leaves of species growing in a box-ironbark forest in southeastern Australia, as well as proportional resorption of nutrients (%). NAS = Nutrient acquisition strategy for each species; references are provided as a footnote. NAS = Nutrient acquisition strategy assigned to each species (N = N-fixing, M = mycorrhizal, H = hemiparasite, C = carnivorous, Pt = proteaceous roots); references are provided as a footnote. Where no nutrient acquisition strategy could be cited, species were assumed to be mycorrhizal.

| **Species** | **NAS** | | **Nitrogen (N)** | | | **Potassium (K)** | | | **Calcium (Ca)** | | | **Magnesium (Mg)** | | | **Phosphorus (P)** | | | **Sulphur (S)** | | |
| --- | --- | --- | --- | --- | --- | --- | --- | --- | --- | --- | --- | --- | --- | --- | --- | --- | --- | --- | --- | --- |
|  |  | | **F** | **S** | **%** | **F** | **S** | **%** | **F** | **S** | **%** | **F** | **S** | **%** | **F** | **S** | **%** | **F** | **S** | **%** |
| *Euryomyrtus ramosissima* | | M^3,4^ | 7150 | 7500 | 4.9 | 4110 | 2620 | -36.3 | 3520 | 4370 | 24.1 | 1830 | 1190 | -35.0 | 334 | 264 | -21.0 | 856 | 979 | 14.4 |
| *Exocarpos cupressiformis* | | H^1^ | 14700 | 10200 | -30.6 | 1620 | 12500 | 671.6 | 5790 | 6020 | 4.0 | 3650 | 3140 | -14.0 | 1540 | 841 | -45.4 | 1530 | 1500 | -2.0 |
| *Grevillea alpina* | | Pt^1^ | 6990 | 6100 | -12.7 | 5600 | 3210 | -42.7 | 3300 | 2580 | -21.8 | 1870 | 1180 | -36.9 | 480 | 279 | -41.9 | 746 | 578 | -22.5 |
| *Hakea decurrens* | | Pt^1^ | 6630 | 1900 | -71.3 | 3010 | 1100 | -63.5 | 1900 | 4360 | 129.5 | 983 | 1490 | 51.6 | 283 | 77 | -72.8 | 943 | 706 | -25.1 |
| *Hibbertia crinita* | M^3^ | | 8730 | 7180 | -17.8 | 6100 | 1850 | -69.7 | 7010 | 6550 | -6.6 | 2010 | 1330 | -33.8 | 414 | 210 | -49.3 | 1960 | 1480 | -24.5 |
| *Hibbertia exutiacies* | M^3^ | | 10500 | 8750 | -16.7 | 5000 | 1750 | -65.0 | 7850 | 7080 | -9.8 | 4220 | 1860 | -55.9 | 495 | 269 | -45.7 | 4660 | 2800 | -39.9 |
| *Leucopogon rufus* | M^1,3^ | | 8370 | 2670 | -68.1 | 2830 | 1530 | -45.9 | 6440 | 7240 | 12.4 | 936 | 894 | -4.5 | 491 | 122 | -75.2 | 1340 | 1290 | -3.7 |
| *Melichrus urceolatus* | M^1^ | | 9760 | 3330 | -65.9 | 3210 | 1600 | -50.2 | 5800 | 4870 | -16.0 | 1280 | 1170 | -8.6 | 670 | 239 | -64.3 | 1140 | 836 | -26.7 |
| *Ozothamnus obcordatus* | M^6^ | | 14100 | 7060 | -49.9 | 13600 | 5340 | -60.7 | 9080 | 11800 | 30.0 | 2270 | 2100 | -7.5 | 664 | 271 | -59.2 | 3230 | 2410 | -25.4 |
| *Philotheca verrucosa* | M^3^ | | 12200 | 3760 | -69.2 | 5410 | 4490 | -17.0 | 6720 | 8510 | 26.6 | 2940 | 3590 | 22.1 | 599 | 179 | -70.1 | 1420 | 1280 | -9.9 |
| *Prostanthera denticulata* | M | | 12100 | 10700 | -11.6 | 5970 | 2840 | -52.4 | 4340 | 4820 | 11.1 | 2010 | 1500 | -25.4 | 588 | 342 | -41.8 | 1390 | 1370 | -1.4 |
| *Pultenaea graveolens* | N^1,2,4^ | | 19700 | 16000 | -18.8 | 4010 | 2240 | -44.1 | 4590 | 6950 | 51.4 | 1810 | 2260 | 24.9 | 347 | 213 | -38.6 | 1670 | 1620 | -3.0 |
| *Pultenaea largiflorens* | N^1,2,4^ | | 8460 | 5280 | -37.6 | 2490 | 1460 | -41.4 | 6710 | 7560 | 12.7 | 1220 | 1060 | -13.1 | 500 | 186 | -62.8 | 1100 | 948 | -13.8 |
| *Stenanthera pinifolia* | M^1^ | | 8460 | 5280 | -37.6 | 2490 | 1460 | -41.4 | 6710 | 7560 | 12.7 | 1220 | 1060 | -13.1 | 500 | 186 | -62.8 | 1100 | 948 | -13.8 |
| *Stypandra glauca* | M | | 14500 | 4960 | -65.8 | 11600 | 3770 | -67.5 | 3260 | 4880 | 49.7 | 1370 | 1280 | -6.6 | 749 | 164 | -78.1 | 1040 | 627 | -39.7 |
| *Xanthorrhoea glauca* | M^3^ | | 6980 | 1660 | -76.2 | 5000 | 1860 | -62.8 | 3220 | 4930 | 53.1 | 1130 | 1620 | 43.4 | 397 | 92 | -76.8 | 711 | 435 | -38.8 |
| **Mean** |  | | **13170** | **7513** | **-41.7** | **6706** | **3726** | **-31.8** | **5420** | **6424** | **27.4** | **2158** | **2088** | **-1.1** | **617** | **224** | **-62.2** | **1436** | **1104** | **-20.0** |

^1^ Pate JS. 1994. The mycorrhizal association: just one of many nutrient acquiring specializations in natural ecosystems. *Plant and Soil* 159: 1–10.

^2^ de Faria SM, Lewis GP, Sprent JI, Sutherland JM. 1989. Occurrence of nodulation in the Leguminosae. *New Phytologist* 111: 607–619.

^3^ Brundrett MC, Abbott L. 1991. Roots of jarrah forest plants. I. Mycorrhizal associations of shrubs and herbaceous plants. *Australian Journal of Botany* 39: 445–457.

^4^ Warcup JH. 1980. Ectomycorrhizal associations of Australian indigenous plants. *New Phytologist* 85: 531–535.

^5^ Kramadibrata, K. 2002. The mycorrhizal status of plants in the Gresswell Nature Reserve, Melbourne, Victoria, Australia. *Berita Biologi* 6: 431–439.

^6^ Warcup JH. 1990. The mycorrhizal associations of Australian Inuleae (Asteraceae). *Muelleria* 7: 179–187.

^7^ Wang B, Qiu YL. 2006. Phylogenetic distribution and evolution of mycorrhizas in land plants. *Mycorrhiza* 16: 299–363.

**Table S1 (contd.).** Concentration of micronutrients (mg^-1^/kg^-1^) in fresh (F) and senesced (S) leaves of species growing in a box-ironbark forest in southeastern Australia, as well as proportional resorption of nutrients (%).

| **Species** | **Boron (B)** | | | **Manganese (Mn)** | | | **Zinc (Zn)** | | | **Copper (Cu)** | | |
| --- | --- | --- | --- | --- | --- | --- | --- | --- | --- | --- | --- | --- |
|  | **F** | **S** | **%** | **F** | **S** | **%** | **F** | **S** | **%** | **F** | **S** | **%** |
| *Acacia acinacea* | 46 | 40 | -13.0 | 137 | 95 | -30.6 | 18.9 | 18.9 | 0.0 | 5.9 | 5.8 | -1.7 |
| *Acacia aspera* | 46 | 51 | 10.9 | 195 | 187 | -4.1 | 22.2 | 17.1 | -23.0 | 5.4 | 3.5 | -35.2 |
| *Acacia genistifolia* | 45 | 58 | 28.9 | 233 | 320 | 37.3 | 16.9 | 16.2 | -4.1 | 5 | 2.8 | -44.0 |
| *Acacia gunnii* | 51 | 55 | 7.8 | 293 | 234 | -20.1 | 13.6 | 26.3 | 93.4 | 5.6 | 2.3 | -58.9 |
| *Acacia montana* | 58 | 48 | -17.2 | 185 | 231 | 24.9 | 17.4 | 23.2 | 33.3 | 6.5 | 2.1 | -67.7 |
| *Acacia paradoxa* | 52 | 41 | -21.2 | 235 | 291 | 23.8 | 17.4 | 13 | -25.3 | 6.7 | 3.4 | -49.3 |
| *Acacia pycnantha* | 46 | 38 | -17.4 | 102 | 115 | 12.7 | 13.8 | 12.3 | -10.9 | 8.2 | 5.6 | -31.7 |
| *Acrotriche serrulata* | 60 | 30 | -50.0 | 268 | 292 | 9.0 | 19.2 | 12.8 | -33.3 | 6.6 | 8 | 21.2 |
| *Amyema miquelii* | 55 | 94 | 70.9 | 145 | 380 | 162.1 | 11.2 | 24.6 | 119.6 | 6.5 | 14.4 | 121.5 |
| *Astroloma humifusum* | 23 | 22 | -4.3 | 170 | 230 | 35.3 | 11.6 | 11.4 | -1.7 | 10.7 | 6.9 | -35.5 |
| *Boronia anemonifolia* | 49 | 53 | 8.2 | 116 | 124 | 6.9 | 20.5 | 27.3 | 33.2 | 4.4 | 3.4 | -22.7 |
| *Brachyloma daphnoides* | 103 | 97 | -5.8 | 268 | 207 | -22.8 | 15.7 | 13.8 | -12.1 | 5.7 | 4.3 | -24.6 |
| *Brunonia australis* | 57 | 56 | -1.8 | 249 | 408 | 63.9 | 105 | 130 | 23.8 | 11.8 | 7.4 | -37.3 |
| *Bursaria spinosa* | 99 | 91 | -8.1 | 939 | 1040 | 10.8 | 103 | 140 | 35.9 | 5.7 | 4.3 | -24.6 |
| *Cassinia arcuata* | 118 | 150 | 27.1 | 893 | 1040 | 16.5 | 107 | 99.6 | -6.9 | 29 | 22.1 | -23.8 |
| *Daviesia leptophylla* | 59 | 56 | -5.1 | 209 | 151 | -27.8 | 33.1 | 21.9 | -33.8 | 5.9 | 2.8 | -52.5 |
| *Daviesia ulicifolia* | 44 | 30 | -31.8 | 185 | 156 | -15.7 | 26.2 | 31.5 | 20.2 | 5.6 | 7.7 | 37.5 |
| *Dianella admixta* | 44 | 30 | -31.8 | 235 | 136 | -42.1 | 74.2 | 31.6 | -57.4 | 5.3 | 3.3 | -37.7 |
| *Dillwynia sericea* | 35 | 23 | -34.3 | 149 | 139 | -6.7 | 20.6 | 17.1 | -17.0 | 4.1 | 3 | -26.8 |
| *Drosera peltata* | 33 | 33 | 0.0 | 52.9 | 89 | 67.3 | 23 | 13.4 | -41.7 | 4.4 | 3 | -31.8 |
| *Eucalyptus goniocalyx* | 104 | 79 | -24.0 | 459 | 436 | -5.0 | 8.8 | 5.8 | -34.1 | 5.2 | 2.6 | -50.0 |
| *Eucalyptus macrorhyncha* | 36 | 44 | 22.2 | 167 | 450 | 169.5 | 7.6 | 6.9 | -9.2 | 4.4 | 2.1 | -52.3 |
| *Eucalyptus melliodora* | 75 | 69 | -8.0 | 444 | 414 | -6.8 | 16.4 | 12.8 | -22.0 | 7 | 4 | -42.9 |
| *Eucalyptus microcarpa* | 57 | 49 | -14.0 | 246 | 485 | 97.2 | 16.4 | 10.7 | -34.8 | 7.4 | 4.6 | -37.8 |
| *Eucalyptus polyanthemos* | 43 | 68 | 58.1 | 271 | 641 | 136.5 | 10.8 | 6.6 | -38.9 | 5.9 | 3.3 | -44.1 |
| *Eucalyptus tricarpa* | 53 | 64 | 20.8 | 166 | 143 | -13.9 | 13.4 | 4.2 | -68.7 | 4.4 | 2.5 | -43.2 |
| *Euryomyrtus ramosissima* | 74 | 55 | -25.7 | 397 | 509 | 28.2 | 8.9 | 9.4 | 5.6 | 3.2 | 3.5 | 9.4 |
| *Exocarpos cupressiformis* | 45 | 36 | -20.0 | 431 | 429 | -0.5 | 12.4 | 19.2 | 54.8 | 12 | 14.3 | 19.2 |
| *Grevillea alpina* | 44 | 18 | -59.1 | 539 | 250 | -53.6 | 7.8 | 4.6 | -41.0 | 2.7 | 1.9 | -29.6 |
| *Hakea decurrens* | 48 | 40 | -16.7 | 197 | 484 | 145.7 | 5.8 | 3.6 | -37.9 | 3.2 | 0.9 | -71.9 |
| *Hibbertia crinita* | 47 | 30 | -36.2 | 201 | 187 | -7.0 | 18.4 | 14.7 | -20.1 | 4.5 | 4.6 | 2.2 |
| *Hibbertia exutiacies* | 78 | 35 | -55.1 | 144 | 162 | 12.5 | 38.2 | 25.2 | -34.0 | 5.9 | 4.9 | -16.9 |
| *Leucopogon rufus* | 22 | 50 | 127.3 | 314 | 335 | 6.7 | 13.4 | 12.7 | -5.2 | 7.9 | 4.4 | -44.3 |
| *Melichrus urceolatus* | 33 | 25 | -24.2 | 542 | 521 | -3.9 | 12.5 | 7.5 | -40.0 | 5.7 | 3.5 | -38.6 |
| *Ozothamnus obcordatus* | 109 | 118 | 8.3 | 1320 | 1720 | 30.3 | 63.5 | 68.5 | 7.9 | 12.8 | 10 | -21.9 |
| *Philotheca verrucosa* | 74 | 111 | 50.0 | 108 | 184 | 70.4 | 10.7 | 7.8 | -27.1 | 6.6 | 3.6 | -45.5 |
| *Prostanthera denticulata* | 92 | 87 | -5.4 | 825 | 751 | -9.0 | 82.5 | 103 | 24.8 | 8.7 | 8.4 | -3.4 |
| *Pultenaea graveolens* | 49 | 59 | 20.4 | 175 | 148 | -15.4 | 29.3 | 32.9 | 12.3 | 5 | 5.1 | 2.0 |
| *Pultenaea largiflorens* | 41 | 40 | -2.4 | 620 | 400 | -35.5 | 13.3 | 8.6 | -35.3 | 7.5 | 5.8 | -22.7 |
| *Stenanthera pinifolia* | 41 | 40 | -2.4 | 620 | 400 | -35.5 | 13.3 | 8.6 | -35.3 | 7.5 | 5.8 | -22.7 |
| *Stypandra glauca* | 42 | 57 | 35.7 | 239 | 273 | 14.2 | 41.1 | 41.7 | 1.5 | 4 | 5.2 | 30.0 |
| *Xanthorrhoea glauca* | 41 | 42 | 2.4 | 61.1 | 138 | 125.9 | 11.9 | 14.7 | 23.5 | 2.6 | 1.4 | -46.2 |
| **Mean** | **57** | **55** | **-0.86** | **327** | **365** | **22.7** | **27** | **27** | **-6.2** | **7** | **5** | **-23.7** |

**Table S2.** The relationship between species rarity and the uniqueness of leaf nutrient profile in senesced leaves and in proportional resorption of nutrients from leaves for 42 species from a box-ironbark forest in southeastern Australia. Linear models were produced considering species frequency within 15 study areas (all sites) in 2010 (pre-burn) and 2013 (post-burn), as well as separately for sites which experienced a burn treatment in 2010 spring autumn, spring, or left unburnt.

| **Burn season** | **Leaf nutrients** | ***F*** | ***P*** | **Adj. *R^2^*** |
| --- | --- | --- | --- | --- |
|  |  |  |  |  |
| ***All sites*** |  |  |  |  |
| Pre-burn | Senesced | 0.03 | 0.87 | -0.02 |
|  | Resorption | 0.16 | 0.69 | -0.02 |
| Post-burn | Senesced | 0.04 | 0.84 | -0.02 |
|  | Resorption | 0.08 | 0.79 | -0.02 |
| ***Unburnt control*** |  |  |  |  |
| Pre-burn | Senesced | 0.04 | 0.85 | -0.02 |
|  | Resorption | 0.03 | 0.86 | -0.02 |
| Post-burn | Senesced | 0.07 | 0.79 | -0.02 |
|  | Resorption | 0.08 | 0.78 | -0.02 |
| ***Autumn burn*** |  |  |  |  |
| Pre-burn | Senesced | 0.02 | 0.88 | -0.02 |
|  | Resorption | 0.22 | 0.64 | -0.02 |
| Post-burn | Senesced | 0.00 | 0.99 | -0.02 |
|  | Resorption | 0.38 | 0.54 | -0.02 |
| ***Spring burn*** |  |  |  |  |
| Pre-burn | Senesced | 0.11 | 0.74 | -0.02 |
|  | Resorption | 0.19 | 0.66 | -0.02 |
| Post-burn | Senesced | 0.11 | 0.74 | -0.02 |
|  | Resorption | 0.34 | 0.56 | -0.02 |

**Table S3.** Eigenvector scores and percent variation explained by five principal components for the similarity of leaf nutrient profiles, and proportional resorption, among 42 evergreen species in a box-ironbark forest in southeast Australia. All ten macro- and micro-nutrients sampled are included in the PCA as eigenvectors. Proportional resorption was calculated as the ratio of nutrients in fresh and senesced leaves.

|  |  | **Senesced** | | | | |  | **Proportional resorption** | | | | |
| --- | --- | --- | --- | --- | --- | --- | --- | --- | --- | --- | --- | --- |
|  |  | **PC1** | **PC2** | **PC3** | **PC4** | **PC5** |  | **PC1** | **PC2** | **PC3** | **PC4** | **PC5** |
| **Percent variation** |  | 36.9 | 16.2 | 12.2 | 10.8 | 8.6 |  | 54.6 | 23.7 | 8.8 | 4.6 | 3.1 |
| **Cumulative variation** |  | 36.9 | 53.1 | 65.3 | 76.1 | 84.7 |  | 54.6 | 78.3 | 87.1 | 91.7 | 94.8 |
|  |  |  |  |  |  |  |  |  |  |  |  |  |
| **Eigenvectors** |  |  |  |  |  |  |  |  |  |  |  |  |
| **B** |  | 0.38 | -0.27 | 0.05 | -0.07 | 0.23 |  | 0.01 | -0.27 | 0.02 | -0.68 | 0.58 |
| **Ca** |  | 0.21 | -0.41 | -0.15 | 0.22 | -0.68 |  | 0.03 | -0.51 | 0.03 | 0.03 | -0.40 |
| **Cu** |  | 0.43 | 0.28 | -0.06 | 0.16 | 0.16 |  | 0.12 | -0.10 | -0.55 | 0.22 | 0.22 |
| **Mg** |  | 0.28 | -0.04 | 0.31 | -0.62 | -0.38 |  | 0.05 | -0.34 | -0.12 | -0.17 | -0.12 |
| **Mn** |  | 0.37 | -0.35 | -0.14 | 0.21 | 0.29 |  | 0.01 | -0.66 | 0.40 | 0.43 | 0.20 |
| **N** |  | 0.02 | 0.44 | -0.54 | -0.43 | -0.08 |  | 0.03 | 0.08 | -0.28 | 0.34 | 0.07 |
| **P** |  | 0.29 | 0.48 | 0.16 | 0.35 | 0.01 |  | 0.06 | -0.06 | -0.30 | 0.26 | 0.19 |
| **K** |  | 0.33 | 0.31 | 0.46 | 0.06 | -0.23 |  | 0.97 | 0.11 | 0.19 | 0.00 | 0.02 |
| **S** |  | 0.32 | 0.07 | -0.55 | 0.12 | -0.21 |  | 0.10 | -0.22 | -0.43 | 0.02 | 0.24 |
| **Zn** |  | 0.35 | -0.17 | -0.02 | -0.41 | 0.36 |  | 0.14 | -0.21 | -0.38 | -0.32 | -0.55 |

**Table S4.** Mean (±SD) concentrations of nutrients (mg^-1^ kg^-1^) in soil samples from a box-ironbark forest in southeastern Australia, collected three years after landscape-scale experimental prescribed burn treatments in autumn, spring, or left as unburnt reference study areas. Samples were taken from upper (0–20 cm) and lower (50–70 cm) soil profiles.

| **Sample** | **Total N** | | **K** | | **Ca** | | **Mg** | | **P** | |
| --- | --- | --- | --- | --- | --- | --- | --- | --- | --- | --- |
| **Upper** |  |  |  |  |  |  |  |  |  |  |
| *Reference* | 831.1 | ± 297.0 | 628.6 | ± 224.9 | 186.7 | ± 155.6 | 344.8 | ± 150.5 | 56.4 | ± 22.3 |
| *Autumn* | 1227.6 | ± 682.2 | 664.8 | ± 284.4 | 256.5 | ± 260.0 | 503.7 | ± 384.9 | 78.0 | ± 31.1 |
| *Spring* | 1127.9 | ± 322.7 | 749.7 | ± 346.7 | 240.8 | ± 141.2 | 560.0 | ± 470.6 | 73.3 | ± 25.0 |
| ***Mean*** | **1108.4** | **± 516.4** | **691.5** | **± 304.9** | **236.3** | **± 201.3** | **494.4** | **± 398.3** | **71.8** | **± 28.3** |
| **Lower** |  |  |  |  |  |  |  |  |  |  |
| *Reference* | 456.3 | ± 119.1 | 935.4 | ± 312.0 | 57.5 | ± 29.6 | 1260.0 | ± 985.7 | 31.8 | ± 12.7 |
| *Autumn* | 438.2 | ± 161.8 | 753.4 | ± 249.7 | 54.4 | ± 32.7 | 1239.8 | ± 1026.2 | 44.7 | ± 32.3 |
| *Spring* | 434.9 | ± 159.3 | 839.9 | ± 320.8 | 93.2 | ± 95.8 | 1174.7 | ± 950.9 | 44.1 | ± 36.1 |
| ***Mean*** | **440.5** | **± 153.4** | **824.4** | **± 300.2** | **70.5** | **± 67.9** | **1217.8** | **± 989.2** | **41.9** | **± 31.6** |
|  |  |  |  |  |  |  |  |  |  |  |
| **Sample** | **S** | | **B** | | **Mn** | | **Zn** | | **Cu** | |
| **Upper** |  |  |  |  |  |  |  |  |  |  |
| *Reference* | 81.2 | ± 27.3 | 13.7 | ± 4.3 | 20.0 | ± 31.6 | 6.5 | ± 4.1 | 5.4 | ± 4.4 |
| *Autumn* | 110.1 | ± 71.3 | 10.3 | ± 2.8 | 19.7 | ± 21.4 | 6.3 | ± 5.8 | 5.7 | ± 3.1 |
| *Spring* | 104.7 | ± 30.1 | 10.2 | ± 4.8 | 21.7 | ± 22.4 | 9.6 | ± 9.2 | 5.7 | ± 2.7 |
| ***Mean*** | **102.2** | **± 51.6** | **10.9** | **± 4.2** | **20.5** | **± 24.2** | **7.6** | **± 7.3** | **5.6** | **± 3.3** |
| **Lower** |  |  |  |  |  |  |  |  |  |  |
| *Reference* | 122.5 | ± 100.9 | 13.0 | ± 4.3 | 31.6 | ± 23.8 | 22.0 | ± 21.1 | 13.9 | ± 7.5 |
| *Autumn* | 131.9 | ± 80.9 | 10.1 | ± 2.3 | 26.2 | ± 53.0 | 18.6 | ± 23.2 | 12.5 | ± 5.7 |
| *Spring* | 139.8 | ± 83.9 | 9.6 | ± 4.4 | 13.0 | ± 14.2 | 24.4 | ± 26.7 | 14.2 | ± 9.6 |
| ***Mean*** | **133.2** | **± 86.7** | **10.5** | **± 3.9** | **20.4** | **± 39.3** | **21.6** | **± 24.4** | **13.5** | **± 7.9** |
